# Real-time single-base specific detection of the *Haemonchus contortus* S168T variant associated with levamisole resistance using loop-primer endonuclease cleavage loop-mediated isothermal amplification

**DOI:** 10.1016/j.mcp.2023.101946

**Published:** 2024-02

**Authors:** Alistair Antonopoulos, Owen Higgins, Stephen R. Doyle, David Bartley, Alison Morrison, Maha Mansour Shalaby, Julien Reboud, Eileen Devaney, Terry J. Smith, Roz Laing, Valentina Busin

**Affiliations:** a School of Biodiversity, One Health and Veterinary Medicine, University of Glasgow, Glasgow, Scotland, United Kingdom; b Kreavet, Kruibeke, Belgium; c Molecular Diagnostics Research Group, School of Biological and Chemical Sciences, University of Galway, Galway, Ireland; d Wellcome Sanger Institute, Hinxton, Cambridgeshire, United Kingdom; e Moredun Research Institute, Penicuik, Scotland, United Kingdom; f James Watt School of Engineering, University of Glasgow, Glasgow, Scotland, United Kingdom; g Food Control Department, Faculty of Veterinary Medicine, Kafrelsheikh University, Kafr-El-Sheikh, Egypt

**Keywords:** LAMP, Resistance, S168T, *Haemonchus contortus*, Veterinary, Levamisole

## Abstract

*Haemonchus contortus* is a parasitic haematophagous nematode that primarily affects small ruminants and causes significant economic loss to the global livestock industry. Treatment of haemonchosis typically relies on broad-spectrum anthelmintics, resistance to which is an important cause of treatment failure. Resistance to levamisole remains less widespread than to other major anthelmintic classes, prompting the need for more effective and accurate surveillance to maintain its efficacy. Loop-primer endonuclease cleavage loop-mediated isothermal amplification (LEC-LAMP) is a recently developed diagnostic method that facilitates multiplex target detection with single nucleotide polymorphism (SNP) specificity and portable onsite testing. In this study, we designed a new LEC-LAMP assay and applied it to detect the levamisole resistance marker S168T in *H. contortus*. We explored multiplexing probes for both the resistant S168T and the susceptible S168 alleles in a single-tube assay. We then included a generic probe to detect the *acr-8* gene in the multiplex assay, which could facilitate the quantification of both resistance markers and overall genetic material from *H. contortus* in a single step. Our results showed promising application of these technologies, demonstrating a proof-of-concept assay which is amenable to detection of resistance alleles within the parasite population, with the potential for multiplex detection, and point-of-care application enabled by lateral flow end-point detection. However, further optimisation and validation is necessary.

## Highlights

• A novel loop-primer endonuclease loop-mediated isothermal amplification assay was developed.
• This proof-of-concept LEC-LAMP assay is the first of its kind to detect the S168T variant associated with levamisole resistance in *Haemonchus contortus*.
• The assay is also amenable to multiplexing probes for targets S168T, S168, and ACR-8.
• Point-of-care end-point detection in the form of a lateral flow assay was also explored.

## 1 Introduction

*Haemonchus contortus* is an economically important haematophagous gastrointestinal nematode (GIN) of small ruminants. If left untreated, infection with *H. contortus* can cause significant morbidity, such as severe anaemia, or death, in the case of a high worm burden. Haemonchosis is also associated with significant production losses in the sheep farming industry globally [1]. Treatment of *H. contortus* infections principally relies on broad-spectrum anthelmintics, the only effective method to clear infection [2]. However, parasite control is seriously threatened by widespread anthelmintic resistance (AR) to all four major classes of anthelmintics: benzimidazoles (BZs); imidazothiazoles such as levamisole (LEV); macrocyclic lactones (MLs); and amino-acetonitrile derivatives (AADs) [[3], [4], [5], [6]], often occurring as multi-drug resistance (MDR) [2,7]. Production losses due to AR are estimated to represent up to €38 million (m) annually in Europe alone [1].

Effective management of AR requires accurate, fast, and readily available diagnostic monitoring. Currently AR diagnosis relies mainly on the faecal egg count reduction test (FECRT) (Kaplan et al., 2023). While this method is conceptually simple, it is labour intensive and lacks sensitivity; for example, BZ resistance may only be detectable when >25 % of the population are resistant [8]. Thus, it is imperative to develop simpler and sensitive diagnostic tools to better manage the use of anthelmintic drugs and control the emergence of AR. Numerous molecular diagnostic tests have been developed for BZ resistance SNPs in parasitic nematodes [[9], [10], [11], [12]], however, most of these assays are best suited to laboratory settings. More recent advances have been made in AR surveillance using high-throughput sequencing of targeted amplicons, for example, using metabarcoding and next-generation sequencing (NGS) of livestock helminths [[13], [14], [15]]. However, this approach typically requires specialised equipment and highly trained staff, in addition to bioinformatic expertise to generate and interpret results, all of which contributes to the high cost, and limited application in the field, of these technologies. There is, therefore, significant scope for alternative technologies to improve the capacity for molecular detection of AR in the field.

Loop-mediated isothermal amplification (LAMP) is a single-temperature nucleic acid amplification method [16] that has been widely used to detect single-cell eukaryotic parasite and helminth DNA. SNP genotyping using LAMP assays has been established successfully for several eukaryotic parasites [[17], [18], [19]]. LAMP assays have also been successfully demonstrated for the identification of individual BZ resistance-associated SNPs in *H. contortus* [[20], [21]]; however, multiplexing several SNPs within a single tube assay has proven difficult using conventional allele-specific (AS) LAMP [20]. Multiplex detection of multiple drug resistance markers has been identified as a necessary step for the adoption of molecular diagnostics to replace the FECRT [2,22]. Loop-primer endonuclease cleavage loop-mediated isothermal amplification (LEC-LAMP) is a real-time, sensitive and specific, novel method that enables multiplex detection with single-base specificity and point-of-use application [23]. LEC-LAMP achieves single base specific cleavage via the inclusion of a loop primer endonuclease IV recognition site flanked by a fluorophore and quencher, enabling fluorescence production after target hybridisation cleavage [23]. LEC-LAMP builds on an earlier technology known as *Tth* endonuclease cleavage (TEC)-LAMP, which functions analogously to LEC-LAMP, however, the endonuclease cleavage recognition site is placed on one of the inner primers, rather than the loop-primer [24]. LEC-LAMP and TEC-LAMP technologies have the potential to bridge the gap between field-based binary detection and multiplexable quantitative laboratory-based technologies such as qPCR and next-generation sequencing [13,25,26]. A recent study demonstrated the applicability of a portable workstation-based LEC-LAMP assay for detecting extended-spectrum beta-lactamase resistance in *E. coli* isolated from porcine faecal samples [27]. Thus, LEC-LAMP may represent a promising tool for detecting AR in the ruminant production sector.

ACR-8, a single subunit of pentameric ligand-gated acetylcholine receptors (AChR), has been shown to play a role in LEV sensitivity [28], while several variants have been proposed to be associated with LEV resistance. We recently identified a single SNP in *acr-8*, encoding an amino acid substitution (S168T), which was strongly associated with LEV resistance [29]. This novel variant was validated using an allele-specific (AS) PCR assay [30] and was subsequently detected in Australian LEV-resistant populations [15] and independently in Swedish field samples [31].

In this study, we have evaluated the use of LEC-LAMP and TEC-LAMP to detect genetic variation associated with levamisole resistance in *H. contortus.* We describe the optimisation and validation of an S168T LEC-LAMP and TEC-LAMP assay to discriminate susceptibility and resistance-associated alleles and explore the adaptation of these assays towards point-of-care detection.

## 2 Materials and methods

### 2.1 Animal handling and ethics statement

All experimental procedures involving animal use were examined and approved by the Moredun Research Institute Animal Welfare Ethical Review Board (AWERB) and were conducted under approved UK Home Office licenses following the Animals (Scientific Procedures) Act of 1986. The Home Office licence number is **P95890EC1**.

### 2.2 *H. contortus* isolates

The *Haemonchus contortus* isolates used in this study were the LEV-susceptible MHco3(ISE) [32,33] and the LEV-resistant MHco18(UGA2004) [34]. All isolates were maintained and passaged using parasite-naive sheep at the Moredun Research Institute, Penicuik, Scotland as described in Doyle et al. [29].

### 2.3 DNA extractions

Crude lysates of single worms were produced using a modified Proteinase K extraction method, as described in Antonopoulos et al. [30]. Using a light microscope, a single L_3_ was added to a well containing 10 μl lysis buffer (10 μl DirectPCR Lysis Reagent (Cell; Viagen 302-C); 0.05 μl 1 M DTT; 0.01 μl 20 mg/ml Proteinase K). Lysates were incubated at 60 °C for 2 h, followed by 85 °C for 45 min to denature the Proteinase K.

### 2.4 Cloning of *acr-8* exon 4 amplicons

To optimise the assay, we used cloned sequences of *acr-8* exon 4 containing either the susceptible (S168) or resistant (S168T) allele. The *H. contortus acr-8* [HCON_00151270; PRJEB506 MHco3(ISE)_4.0 assembly [35]] exon 4 and a ∼20–25 bp region of intron 5 were amplified, using MHco18(UGA2004) for both the resistance associated S168T, and susceptible associated S168 allele, by Phusion Green HiFi PCR with primers Hco-Intron4-F and Hco-exon4-R as described in Antonopoulos et al. [30]. The MHco18(UGA2004) isolate was chosen as it is a heterogeneous population, with both homozygous S168 and homozygous S168T individuals, in addition to heterozygotes. Individuals homozygous for S168T or S168 were then identified by S168T AS-PCR [30], from which a single S168 allele was selected from MHco3(ISE) individual L_3_ and a single S168T allele was selected from MHco18(UGA2004) individual L_3_. S168 and S168T exon 4 fragments were PCR purified using QIAQuick PCR Purification Kit (Qiagen, 28104). Purified S168T and S168 exon 4 fragments were then cloned using TOPO™ TA Cloning™ (Invitrogen, K450001) with XL-10 gold ultra-competent *E. coli* cells (Agilent, 200315) according to the manufacturer's instructions. TA tailing was carried out using *Taq* DNA polymerase (NEB, M0273S). Colonies were cultured on LB-agar with 100 μg/ml ampicillin (Sigma Aldrich, A9518). Colonies were picked and screened for the presence of the correct insert by S168T AS-PCR and screened for SNPs at the primer binding site by Sanger Sequencing [30]. Colonies containing the S168T or S168 allele were subcultured onto LB-agar with 100 μg/ml ampicillin. Plasmids containing either the S168T or S168 allele were extracted and concentrated via mini-prep using the QIAprep Spin Miniprep Kit (Qiagen 27104). DNA concentrations were estimated using a Qubit spectrophotometer (Thermofisher Scientific) using the Qubit dsDNA Broad Spectrum assay kit (Thermfisher Scientific, Q33230), after which the concentration was adjusted to ∼20 ng/μl. A high initial concentration of DNA material was chosen to ensure good amplification was achieved.

### 2.5 LAMP

#### 2.5.1 LAMP assay design

Whole-genome sequencing data from pools of 200 MHco3(ISE), MHco18(UGA2004), and MHco3/18 L_3_ [29] were aligned to the MHco3(ISE) reference sequence [PREB506.WBPS13 WormBase ParaSite annotation, PRJEB506 MHco3(ISE)_4.0 assembly [35]]. Reads aligned to the *acr-8* genomic locus [HCON_00151270] were visually inspected in Geneious (Biomatters Ltd: 11.1.5) to identify inter-isolate polymorphisms and generate a consensus sequence. Consensus sequences were then exported from Geneious and uploaded to the Primer Explorer V5 web application (https://primerexplorer.jp/e/; Eiken Chemicals, Japan) initially under default parameters to generate candidate primer sequences which were then edited manually to optimise primer melting temperature (T_m)_ values, avoiding overlap between the B1 and B2c primer binding sites, and ensuring a T_m_ of 66–68 °C for the loop primers. Final primer sequences are summarised in Supplementary Table 1.

#### 2.5.2 LEC-LAMP assay design

The LEC-LAMP assay was designed following guidelines per Higgins and Smith [23], avoiding overlap of primer binding sites between B1 and B2c regions where possible, and a preference for T_m_ values of between 6668 °C (Fig. 1). LEC-LAMP probes were designed manually based on binding sites for LAMP primers (described in section 2.4) and were sited between B1 and B2c, with the S168T mutation-specific binding site located between the 5’ BHQ1 and FAM tags. The S168T mutation-specific binding site is flanked by a basic site adjacent to the FAM tag. Overlap with B1 was avoided; however, some overlap with B2c was unavoidable. Probes were designed with a preference for T_m_ values of ∼66 °C to increase binding specificity. Two probes were designed, each specific to either the resistant or susceptible (S168T/S168) binding sites (Supplementary Table 1). A modified fluorescent conjugated TEC-LAMP BIP probe was also designed following Higgins et al. [24], targeting the *acr-8* exon 4 locus to serve as a generic *H. contortus* detection probe. This probe is hereafter referred to as a TEC-LAMP probe, so as to differentiate it from the tagged loop-primer used in LEC-LAMP. However, unlike the TEC-LAMP described by Higgins et al. [24], *Tth* endonuclease IV is not used in this study as endonuclease IV. All probes were synthesised by Metabion AG, Munich, Germany. All LAMP primers were synthesised by Integrated DNA Technologies (IDT) BVBA, Leuven, Belgium. All reactions were carried out a minimum of three times to ensure validity of the result.

**Fig. 1:**
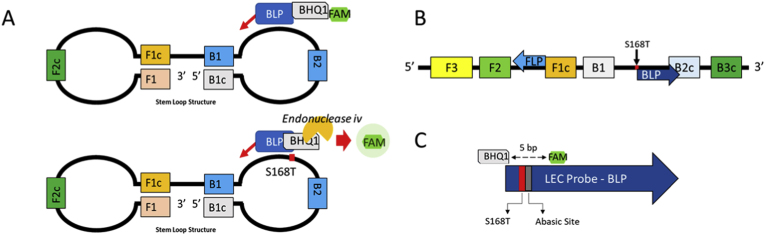
A: Schematic representation of LEC-LAMP assay design. The loop structure formed during LAMP amplification contains the site for the S168T/S168 SNP, which in turn forms the binding site for the loop primer. Once the loop primer binds to the amplicon loop, the abasic site flanking the base matching the S168T/S168 SNP on the loop primer forms the cleavage recognition site for *endonuclease* IV, triggering enzymatic cleavage and liberation of the fluorophore from the quencher moiety. The presence a SNP in the probe target area (a base mismatch between the probe and target) will cause imperfect binding and thus no cleavage or fluorescence. If the probe is a perfect match for the allele SNP target, then there is no SNP in relation to the probe, and thus cleavage will occur. B: Schematic representation of primer binding sites. Loop primers are indicated by arrows. BLP: Backward loop primer. FLP: Forward loop primer. C: Schematic representation of LEC-LAMP probe design.

#### 2.5.3 LEC-LAMP assay conditions

LEC-LAMP reactions were set up as follows: 1.6 μmol/l FIP/BIP, 0.4 μmol/l LEC primer/probe LF/LR, and 0.2 μmol/l F3/B3, 8 U *Bst* 2.0 WarmStart DNA polymerase (New England Biolabs), 1 U endonuclease IV (New England Biolabs), 1 μl DNA template, or 1 μl molecular-grade water for no template control (NTC) [23] in a final LAMP assay reaction volume of 25 μl. 1 μl of cloned S168T or S168 exon 4 fragments was used as a DNA template for LEC-LAMP. Reaction temperatures from 61 to 68 °C were trialled. LAMP reactions were validated using the NEB LAMP fluorescent dye (B1700S) as a positive control for DNA amplification. FAM channel (susceptible – S168 probe) and HEX channel (resistant – S168T probe) fluorescence detection on the Mx3001 qPCR machine was trialled at probe concentrations of 0.4–0.8 μM. ROX (Qiagen) was used as reference dye for all qLAMP reactions. Fluorescence analysis for all LEC-LAMP reactions was carried out in MxPro V7 qPCR software (Agilent). All fluorescence measurements were taken using the dRn, or normalised reporter value, the Rn value minus the baseline Rn value generated by the reporter dye (ROX). This was chosen as it reliably calculates the magnitude of the specific signal generated by the reaction taking account of the baseline fluorescence within the reaction. The baseline threshold for detection was set at 0.5 dRn, which was chosen based on experimental data during optimisation.

#### 2.5.4 Multiplex LEC-LAMP

LEC-LAMP reactions were set up as described in 2.5.3, except using duplex probe 10X LAMP primer stock solution (Supplementary Table 1). FAM channel (susceptible – S168 probe) and HEX channel (resistant – S168T probe) fluorescence detection on Mx3001 qPCR machine was trialled at probe concentrations of 0.8 μM. Triplex LEC-LAMP reactions were set up as described in 2.5.3, except using triplex probe 10X LAMP primer stock solution (Supplementary Table 2) incorporating 0.2 μM Cy5 conjugated BIP. ROX (Qiagen) was used as a reference dye for all qLAMP reactions. The dRn measurement was used for multiplex LEC-LAMP reactions as described in 2.5.3.

### 2.6 Lateral flow LEC-LAMP

#### 2.6.1 Assay design

**Fig. 2:**
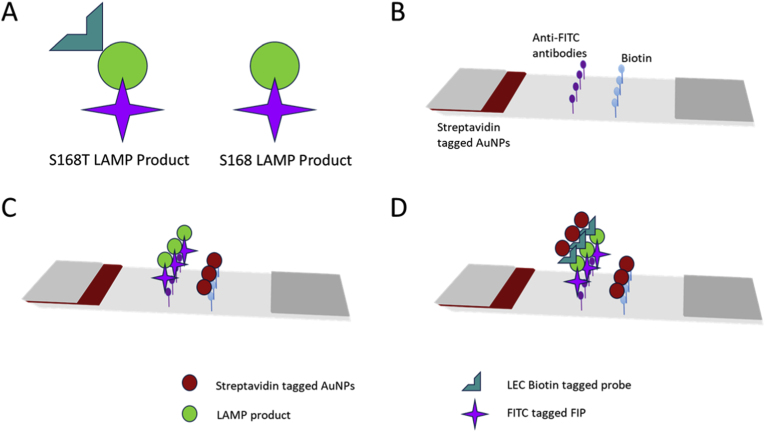
Schematic representation of the initial S168T specific lateral flow LEC-LAMP assay design. A: Schematic representation of the FIP and BLP probes used for LEC-LAMP lateral flow. The LEC BLP is tagged with Biotin, and the FIP with FITC. The amplicon formed thus has both tags, if S168T is present. If S168T is not present, the amplicon will be cleaved, eliminating the biotin tag. B: Schematic representation of the lateral flow strip. The reaction mixture is pipetted into the sample well, whereupon it flows past the streptavidin tagged AuNPs, followed by the anti-FITC test line, and finally the biotin control line. C: Schematic representation of a negative result on the lateral flow strip indicating the absence of the S168T variant. LAMP amplicons are immobilised on the anti-FITC test line, however, streptavidin tagged AuNPs do not bind, as the biotin tag has been cleaved from the amplicon. AuNPs are immobilised by biotin, leading to a colour change on the control line, indicating the test functioned. D: Schematic representation of a positive result on the lateral flow strip indicating the presence of the S168T variant. LAMP amplicons are immobilised on the anti-FITC test line, streptavidin tagged AuNPs bind to the biotin tag, leading to a colour change on the test line. AuNPs are immobilised by biotin, leading to a colour change on the control line, indicating the test has functioned correctly. (For interpretation of the references to colour in this figure legend, the reader is referred to the Web version of this article.)

Adaptation of the LEC-LAMP probe to lateral flow was achieved by conjugation with a fluorescein isothiocyanate (FITC) moiety at the 5’ end of the FIP and the conjugation of the BLP with a biotin moiety. This forms a FITC-biotin conjugated amplicon in the presence of the S168T allele, which is then detectable on the lateral flow strip. As a result, without the S168T allele, the FITC moiety is cleaved from the probe, leading to a biotin-conjugated amplicon that will not aggregate with anti-FITC antibody immobilised on the test line of lateral flow strip, and as a result, no colour change would be detected (Fig. 2).

#### 2.6.2 Lateral flow LEC-LAMP reaction

The lateral flow LEC-LAMP reaction was carried out as described for 2.5.3, except with the inclusion of 0.004 μM lateral flow LEC-LAMP probe and 0.008 μM FITC labelled FIP. Then, following the reaction, 5 μl of the LEC-LAMP products, including the labelled lateral flow probe and FITC labelled FIP, were pipetted directly onto the lateral flow strip (Disposable Nucleic Acid Detection Strip, D003-03, Ustar Biotechnologies, Hangzhou, China). The lateral flow strip was left at room temperature for 15 min before the result was interpreted as described in the manufacturer's instructions.

## 3 Results

### 3.1 LEC-LAMP

#### 3.1.1 Singleplex LEC-LAMP

**Fig. 3:**
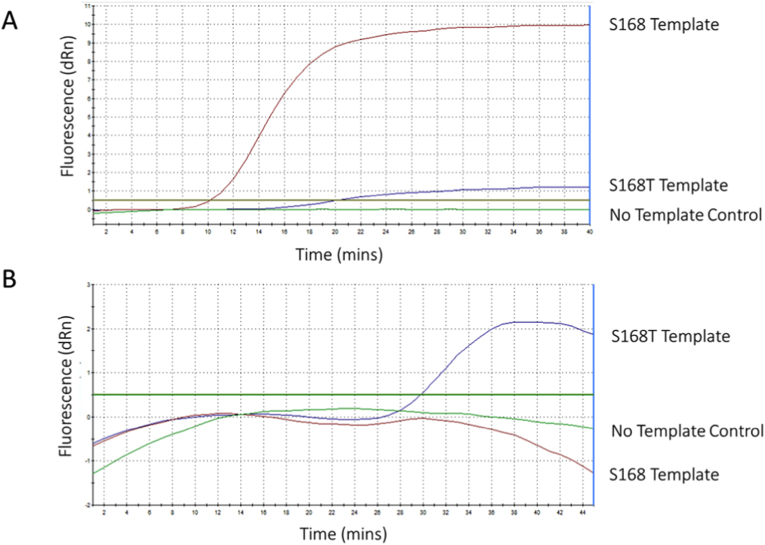
Singleplex allele-specific LEC-LAMP identification of *H. contortus* S168 and S168T alleles. Each probe was challenged with either the S168T or the S168 allele within a single-tube reaction. Each curve represents a single tube reaction. A: S probe challenged with S168T or S168 template. B: R probe challenged with S168T and S168 template. The threshold for detection was set to 0.5 dRn based on experimental data during optimisation.

Initial optimisation of the LEC-LAMP assay showed allele-specific discrimination when using the LEC-LAMP S168 (S) probe challenged individually with the S168 or the S168T template (Fig. 3 A). Allele-specific discrimination was also achieved with the LEC-LAMP S168T (R) probe when challenged with either the S168T or S168 template (Fig. 3 B). The R probe, however, did not maintain constant cleavage with the same efficiency as the S probe, indicated by the drop in relative fluorescence seen following the initial plateau (Fig. 3 B).

#### 3.1.2 Multiplex LEC-LAMP

**Fig. 4:**
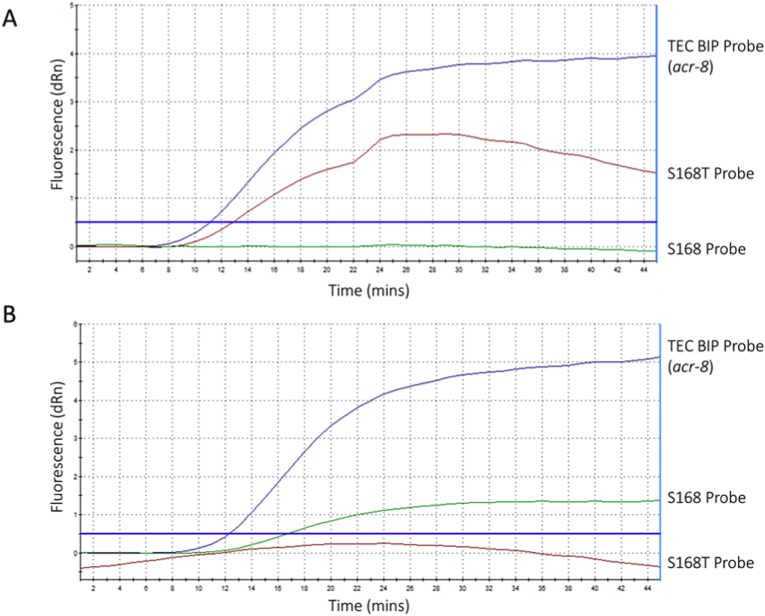
Multiplex S168T, S168, and *acr-*8 LEC-LAMP challenged with single sample templates. Multiplex LEC-LAMP for the simultaneous detection of the *acr-8* gene, S168T and S168 alleles within a single tube assay at 63 °C using generic *acr-8* probe, susceptible (S) and resistant probe (R). A: Detection of S168T allele and *acr-8* in a single tube assay by a modified resistant probe (R) and TEC-BIP probe challenged with the S168T template. B: Detection of the S168 allele and *acr-8* in a single tube assay by the susceptible probe (S) and TEC-BIP probe challenged with the S168T template. The threshold for detection was set to 0.5 dRn based on experimental data during optimisation.

Following this, a triplex LEC-LAMP reaction incorporating a fluorescent conjugated BIP probe targeting the *acr-8* gene alongside the R and S probes was challenged with either the S168T or the S168 template (Fig. 4). Detection of the *acr-8* gene was observed in both reactions challenged with each template (Fig. 4 A, B). An improvement in the fluorescence signal of the R probe was also observed when challenged with the S168T template in the triplex reaction (Fig. 4 A) when compared to the singleplex assay (Fig. 3 B). In contrast, the S probe in the triplex assay showed much weaker overall fluorescence (Fig. 4 B) compared to its fluorescence in the singleplex assay (Fig. 3 A).

**Fig. 5:**
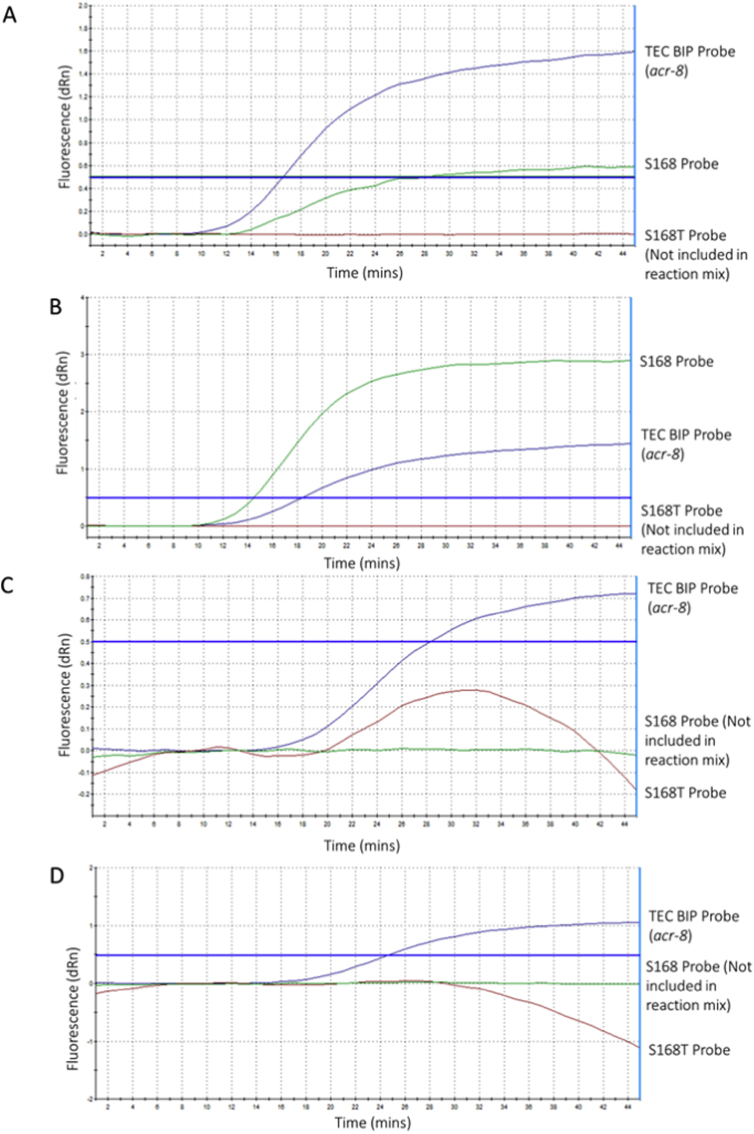
Duplex two tube S168T/S168, and *acr-*8 LEC-LAMP challenged with mixed sample templates. Duplex LEC-LAMP for the simultaneous detection of the *acr-8* gene, S168T or S168 alleles within a single tube assay at 63 °C using generic *acr-8* probe, susceptible (S) and resistant probe (R). A: Detection of the S168 allele and *acr-8* in a single tube assay by the susceptible probe (S) and LEC-BIP probe challenged with a 2:1 mix of the S168T:S168 templates. B: Detection of S168 allele and *acr-8* in a single tube assay by the susceptible probe (S) and LEC-BIP probe challenged with a 1:2 mix of the S168T:S168 template. C: Detection of S168T allele and *acr-8* in a single tube assay by the modified resistant probe (R) and LEC-BIP probe challenged with a 2:1 mix of the S168T:S168 template. D: Detection of S168T allele and *acr-8* in a single tube assay by the modified resistant probe (R) and LEC-BIP probe challenged with a 1:2 mix of the S168T:S168 template. The threshold for detection was set to 0.5 dRn based on experimental data during optimisation.

When the triplex assay was challenged with both the S168T and S168 templates in a single tube, the assay performed poorly (Supplementary Fig. 1). Thus, finally we trialled the assay as a duplex two-tube assay to detect the S168T or S168 allele and the *acr-8* gene, to determine if including only a single allele-specific probe would improve performance when challenged with a mixed sample containing both allele templates. Each duplex reaction was challenged with two mixes (1:2 or 2:1) of S168T and S168 (Fig. 5) to determine if a proportional increase or decrease in fluorescence would be detectable. The results showed that the generic *acr-8* targeting BIP probe continued to perform well across all samples, with a significant improvement in detecting the allele of interest using the susceptible probe (S). Furthermore, the susceptible (S) probe showed a marked increase in overall fluorescence when the proportion of the S168 allele was increased in the sample (Fig. 5 A, B), while the generic *acr-8* targeting BIP probe fluorescence remained stable across both samples. The modified resistant (R) probe, however, showed some differentiation between the alleles of interest in the 2:1 mix of S168T:S168 (Fig. 5C), but not in the 1:2 mix of S168T:S168 samples (Fig. 5 D), and failed to cross the threshold for detection in either, although a sigmoidal curve indicating exponential increase in cleavage in line with LAMP amplification was seen in the 2:1 S168T:S168 reaction (Fig. 5C).

### 3.2 Lateral flow LEC-LAMP

**Fig. 6:**
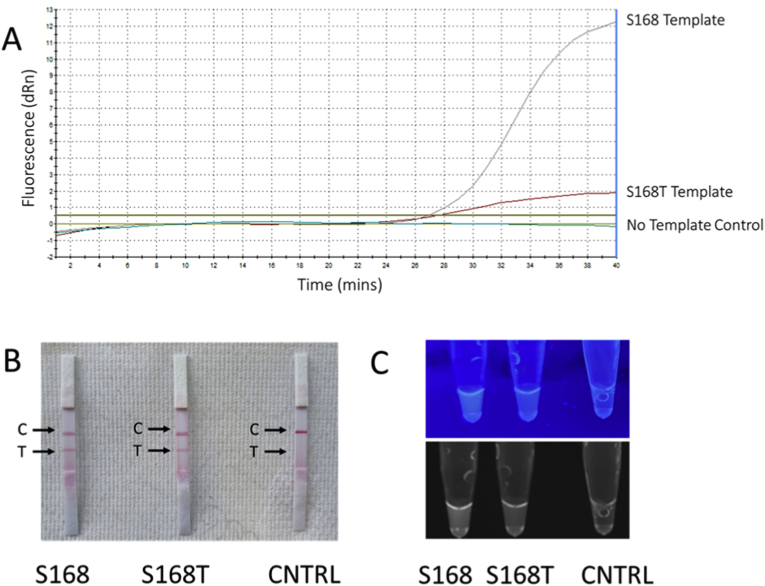
Evaluation of two different point-of-care amenable end-point detection methods for LEC-LAMP detecting the S168 allele using cloned *H. contortus acr-8* exon 4 fragment plasmid. A: Fluorescent LEC-LAMP showing discrimination via fluorescence between S168T and S168 allele by the susceptible probe (S). B: Lateral-flow LEC-LAMP by lateral-flow modified biotin labelled susceptible probe (S) and FITC labelled BIP primer. S168: S168 allele plasmid. S168T: S168T allele plasmid. CNTRL: no template control. C – control line: indicates that the lateral-flow assay is functional and that AuNPs have bound to the control line. T – test line: indicates that the amplicon has been detected. C: Naked eye fluorescence discrimination between the S168T and S168 alleles using LEC-LAMP S probe. Top image: UV light box. Bottom image: UV gel doc (UVITEC Firereader). The same samples were used for all images in this figure. The threshold for detection was set to 0.5 dRn based on experimental data during optimisation.

Developed reactions for the S168T LEC-LAMP were then transferred to lateral flow end-point detection (Fig. 6). Reaction performance in the presence of the lateral flow-modified primers was initially assessed using the fluorescent S probe to ensure that conjugation with FITC and biotin did not negatively impact the reaction overall. However, the LF probe produced detectable fluorescence higher than the threshold of detection significantly later (cycle 27) (Fig. 6 A) and the S probe (cycle 12) (Fig. 3 A). The lateral flow assay was not allele-specific, as a colour change was seen on the test (T) line for both the S168T and the S168 alleles (lateral flow dip sticks 1 and 2; Fig. 6 B). No unspecific amplification was observed, as there was not any colour change on the test line of the lateral flow strip in the negative control sample (no template control sample). (lateral flow dip stick 3; Fig. 6C). This indicated that the lateral flow LEC-LAMP assay serves as detection for the *acr-8* gene. Naked eye fluorescence detection under UV light was also examined, but, the difference in fluorescence observed between the S168T and S168 samples was insufficient for clear allele-specific discrimination (Fig. 6C).

## 4 Discussion

In this study, we designed and tested a multiplex LEC-LAMP assay to detect the recently described S168T SNP associated with LEV resistance in *H. contortus* [15,[29], [30], [31]]. Two probes were designed and tested for detecting and discriminating between the S168T and S168 variants in a fluorescent LEC-LAMP assay, with a third probe designed to target a conserved site of the *acr-8* exon 4 region and act as a benchmark against which allele frequency could be determined within a population. Assay performance when challenged with both alleles simultaneously in this format was not optimal, with allele specific discrimination not possible. Consequently, the assay was modified to a two-tube duplex, combining the S168T or S168 probe with the *acr-8* gene probe and challenged with both alleles simultaneously, highlighting detectable differentiation between each allele in a mixed sample with the S probe, however, the assay continued to perform poorly in this format with the R probe. Finally, we evaluated the combination of LEC-LAMP with lateral flow end-point detection, which could accurately detect the *acr-8* gene, but could not differentiate between the S168T and S168 alleles.

A significant advantage of the LEC-LAMP technology is the use of fluorescence for detection, rather than turbidity or colour change commonly used in LAMP assays for SNP genotyping [[17], [18], [19]]. Fluorescence detection is readily amenable to quantification in the field, due to recent advances in smartphone-based technology [36]. In addition, while there is already an AS-PCR which has been developed for detecting the S168T variant [30], it is worth noting that this AS-PCR is only suitable in a laboratory setting, as it makes use of gel electrophoresis for end-point detection. Although the S168T PCR has performed well and been used for genotyping of field populations, the protocol is relatively labour intensive and requires specialised equipment. This is one of the principal motivations for the development of the S168T LEC-LAMP, with a view to developing an assay which would be point-of-care amenable and potentially useable by veterinarians in the field. This study represents the first demonstration of the adaptability of LEC-LAMP to eukaryotic parasites, in addition to illustrating the first example of the adaptation of a SNP-specific enzymatic cleavage-based fluorescence LAMP method to *H. contortus*. Our LEC-LAMP assay is also amenable to multiplexing, as demonstrated by including two different probes, one targeting the *acr-8* gene and the other the S168 allele. Although previous LAMP assays for *H. contortus* have targeted the ITS2 region (Melville et al., 2014; Khangembam et al., 2021), this was not judged to be suitable for the purposes of this study. This is primarily due to the fact that the inclusion of the *acr-8* probe was chosen to facilitate future quantification of resistance alleles within a population. The ITS2 region is a conserved multi-copy locus (Redman et al., 2008 [30]), which, although of great utility for species identification, would not be useful for benchmarking levamisole resistance alleles due to a differing copy number to *acr-8*. When trialling the triplex reaction format incorporating the *acr-8* specific probe, the R probe performed poorly, requiring further optimisation to maintain the same level of reaction efficiency as in singleplex format. In addition, our inclusion of a TEC-LAMP probe by modifying the inner LAMP primer [24], could serve as an internal control target, which would allow for the incorporation of internal reaction validation in future work. Finally, this represents the first example of combining the TEC-LAMP [24] and LEC-LAMP [23] technologies in a single assay, which further advances the field of molecular diagnostics in general. Further improvements moving forward however, are necessary. Our input concentration was high, 20 ng, which was done to ensure good amplification during the optimisation process, and to demonstrate the amenability of the technology to the detection of S168T. In future, however, it would be beneficial to reduce this, which will require further assay optimisation. In addition, it would be beneficial in future work to explore the sequence diversity of *acr-8* sequences in related GINs of veterinary importance, to determine if the primers used herein showed any cross-reactivity with closely related nematodes such as *Teladorsagia circumcincta*. Given an analogue of the S168T variant has been detected in *T. circumcincta acr-8*, this could open the possibility of a multi-species test. However, further work is necessary to clarify this.

To develop these assays further there are still significant barriers to overcome. Multiplexing detection for several alleles, and direct detection of worm egg DNA from faeces, has long presented a challenge for developing and adopting LAMP for SNP genotyping [20]. In the assay developed herein, we sought to offset previously encountered difficulties multiplexing several different primer sets in a single assay [20] by using SNP-specific fluorescence detection rather than amplification refractory allele-specific primer design. However, this presented its own unique set of challenges related to probe optimisation. Both probes target the same locus and are, in effect, modified reverse loop primers, raising the potential issue of competitive binding at the SNP locus. This is further compounded by the fact that cleavage will only occur in the case of an allele-specific match at the SNP locus, but this will not prevent the binding of the probe. Thus, if the binding affinity is higher for one probe, then this can competitively block the binding of the other probe, potentially lowering the overall fluorescence signal, as was seen when the triplex reaction was challenged with mixed samples (Supplementary Fig. 1). Therefore, we tested the assay as a duplex *acr-8* and S168T/S168 two-tube assay. This resolved the issue of competitive binding for the S probe but did not improve the detection of the S168T allele with the R probe. This raises an important issue that must be overcome before this technology can be adapted to multi-drug resistance detection. We also demonstrated a relationship between the fluorescence intensity of the allele-specific probe and the quantity of the allele within a sample for the S probe, but this was not possible with the R probe. In contrast, the fluorescence signal for *acr-8* remained relatively constant across all samples. The assay limitations encountered in this study could be overcome with further assay and reaction optimisation in future work, but these lay beyond the scope of the current study to develop a proof-of-concept assay for LEV resistance marker detection. Finally, the current assay was only tested on cloned alleles from single *H. contortus* L_3_. Therefore, future work should also focus on mixed samples from pooled L_3_ larvae to determine how the assay performs on polymorphic samples.

Finally, we sought to explore the amenability of the LEC-LAMP assays developed in this study to lateral flow and naked-eye fluorescence detection. LEC-LAMP unfortunately did not prove to be amenable for naked-eye fluorescence detection under the current reaction conditions. An increase in probe concentration might improve this, however, the cost of the probes prohibits this from being a useful solution for point-of-care tests. Colour change or turbidity remains the optimal method for naked eye detection, however, this is not practicable for multiplex SNP genotyping. We thus decided to focus efforts on lateral flow detection. A significant advantage of lateral flow detection is the simplicity of use, as demonstrated by its widespread adoption for self-testing during the SARS-CoV-2 pandemic. Demonstrating the amenability of LEC-LAMP to lateral-flow detection also represents the significant translational potential for multiplexed detection of resistance-associated SNPs. Initial experiments demonstrate that LEC-LAMP is also amenable to end-point detection; however, this technology requires further work to achieve SNP specificity. Our initial lateral-flow assay focused on detecting the S168T allele, however, due to a limitation in our design, we could only confirm the presence (or absence) of the *acr-8* exon 4 fragment. This is likely due to the detection of uncleaved rather than cleaved products. This could, nonetheless, serve as a screening test to detect infection with *H. contortus* in the field. Though there are already LAMP assays for the detection of haemonchosis (Melville et al., 2014; Khangembam et al., 2021) these both target the ITS2 region, and thus, are not suitable for the detection of levamisole resistance. However, it may be of benefit in future studies to explore the combination of ITS2 and *acr-8* primer sets to combine species specific detection with levamisole resistance detection. As cleavage only occurs in the presence of a match, the susceptible probe was modified for lateral-flow end-point detection. This would, thus, yield an uncleaved product if the S168T allele was present, allowing for detection of the uncleaved product corresponding to the lateral flow probe remaining bound to the allele of interest with both moieties intact, hybridised to the lateral-flow strip. However, this assumes that 100 % of the bound probe will be cleaved by the *endonuclease* IV enzyme within the time of the reaction (40 min). Thus, a future improvement to the assay should take this into account. The specificity may also be improved by including a second label to the LEC-LAMP probe, allowing for the detection and differentiation of both cleaved and uncleaved amplicons. This could also serve a dual purpose, allowing for simultaneous species-specific detection in addition to detecting the S168T allele. Nevertheless, our demonstration of the amenability of the LEC-LAMP technology to rapid point of care-based end-point detection represents a significant step forward for the field of veterinary parasitology and anthelmintic resistance detection by contributing a multifunctional and low instrumentation requirement proof-of-concept assay.

## 5 Concluding remarks

In this study, we evaluated novel LEC-LAMP technology for detecting the S168T allele in *H. contortus*. We developed a multiplex LEC-LAMP assay which showed clear allele-specific discrimination between the S168T and S168 alleles using cloned sequences, demonstrating the efficacy of this approach in a proof-of-concept assay. We also included an assay targeting a conserved region of the *acr-8* gene which could be used as a baseline for quantifying DNA within the sample. This multiplex LEC-LAMP assay serves as a proof-of-concept demonstration of the amenability of enzymatic cleavage-based LAMP for detecting anthelmintic resistance in *H. contortus* and constitutes an important step towards realising a multiplex, point-of-care molecular diagnostics to detect anthelmintic resistance. Finally, we demonstrated that the TEC-LAMP and LEC-LAMP technologies could be combined into a single functional assay, with significant implications for the field of molecular diagnostics in general.

## CRediT authorship contribution statement

**Alistair Antonopoulos:** Conceptualization, Investigation, Methodology, Project administration, Resources, Software, Validation, Visualization, Writing – original draft, Writing – review & editing. **Owen Higgins:** Conceptualization, Investigation, Methodology, Resources, Validation, Visualization, Writing – review & editing. **Stephen R. Doyle:** Conceptualization, Investigation, Methodology, Writing – review & editing. **David Bartley:** Resources, Writing – review & editing. **Alison Morrison:** Resources, Writing – review & editing. **Maha Mansour Shalaby:** Conceptualization, Investigation, Methodology, Resources, Validation, Visualization, Writing – review & editing. **Julien Reboud:** Investigation, Methodology, Resources, Writing – review & editing. **Eileen Devaney:** Conceptualization, Funding acquisition, Investigation, Methodology, Project administration, Resources, Supervision, Visualization, Writing – original draft, Writing – review & editing. **Terry J. Smith:** Supervision, Writing – review & editing. **Roz Laing:** Conceptualization, Funding acquisition, Investigation, Methodology, Project administration, Resources, Supervision, Visualization, Writing – original draft, Writing – review & editing. **Valentina Busin:** Conceptualization, Funding acquisition, Investigation, Methodology, Project administration, Resources, Supervision, Visualization, Writing – original draft, Writing – review & editing.

## Figures and Tables

**figs1:**
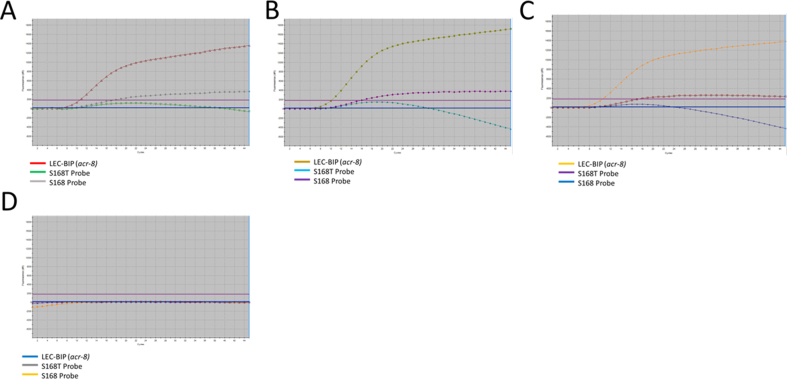


## Data Availability

Data will be made available on request.
