# Supplementary material for: Real-time single-base specific detection of the Haemonchus contortus S168T variant associated with levamisole resistance using loop-primer endonuclease cleavage loop-mediated isothermal amplification
Source: Mol Cell Probes. 2024 Feb;73:None. doi: 10.1016/j.mcp.2023.101946 (PMC10884526; doi:10.1016/j.mcp.2023.101946)
Supplement: Multimedia component 1 [file mmc1.docx]

Supplementary Materials

[Supplementary Table 1: LAMP and PCR oligonucleotides.]

| **Primer Name** | **Target** | **Sequence (5**′**-3**′**)^a,b^** | |
| --- | --- | --- | --- |
| Hco-Intron4-F | *acr-8* exon 4 | GTGATTTCGTGCAGAGATAGG | |
| Hco-exon4-R | *acr-8* exon 4 | AATTATGAGCGATGCCCTC | |
| F3 | *acr-8* exon 4 | CTATCTATGTGATTTCGTGCAG | |
| B3 | *acr-8* exon 4 | CATTCTGTCCAATCAATATCGAG | |
| FIP | *acr-8* exon 4 | CGGTAAGTTTGGTGGTCTCTGGAATAGGAGAAGTGGCGCATT | |
| BIP | *acr-8* exon 4 | GCTGCCGCACATCTAAGAGGAATCGTATGATTCTCAGTCATGCTC | |
| LF | *acr-8* exon-4 | ACTGTTGAAAACTTTCTACCAATAAAGA | |
| LEC-LAMP (R)^a, b^ | S168T allele | BHQ1-TGTA(dSpacer)**G**(FAM-dT)CCATCCTCCAAATTTTAAAGAGC | |
| LEC-LAMP (S)^a, b^ | S168 allele | BHQ1-TGTA(dSpacer)**C**(FAM-dT)CCATCCTCCAAATTTTAAAGAGC |  |
| TEC-LAMP BIP (*acr-8*) | *acr-8* exon 4 | BHQ2-GCTG(dspacer)CG(Cy5-dC)ACATCTAAGAGGAATCGTATGATTCTCAGTCATGCTC | |
| LEC-LAMP LF (R)^a, c^ | S168 allele | Biotin- TGTA(dSpacer)CTCCATCCTCCAAATTTTAAAGAGC | |

a: (dSpacer) denotes abasic cleavage recognition site for Endonuclease IV

b: BHQ1 denotes black hole quencher 1, FAM denotes carboxyfluorescein fluorophore

c: FITC denotes fluorescein-5-isothiocyanate

**Bold underlined** bases identify SNP

[Supplementary Table 2: 10X LAMP primer stock solution.]

| **Primer** | **1X final concentration (25 μl)** | **Volume 100 pmol primer stock to make 10X solution (125 μl)** |
| --- | --- | --- |
| FIP | 0.8 μM | 10 μl |
| BIP | 0.8 μM | 10 μl |
| F3 | 0.2 μM | 2.5 μl |
| B3 | 0.2 μM | 2.5 μl |
| FLP^a^ | 0.4 μM | 5 μl |
| BLP^a, b^ | 0.4 μM | 5 μl |
| DNAse/RNase-free water^a^ | n/a | 90 μl |

a: Concentration and final volume are adjusted accordingly when LEC-LAMP probe is substituted for a loop primer

b: or LEC-LAMP probe (concentration range 0.8 μM)


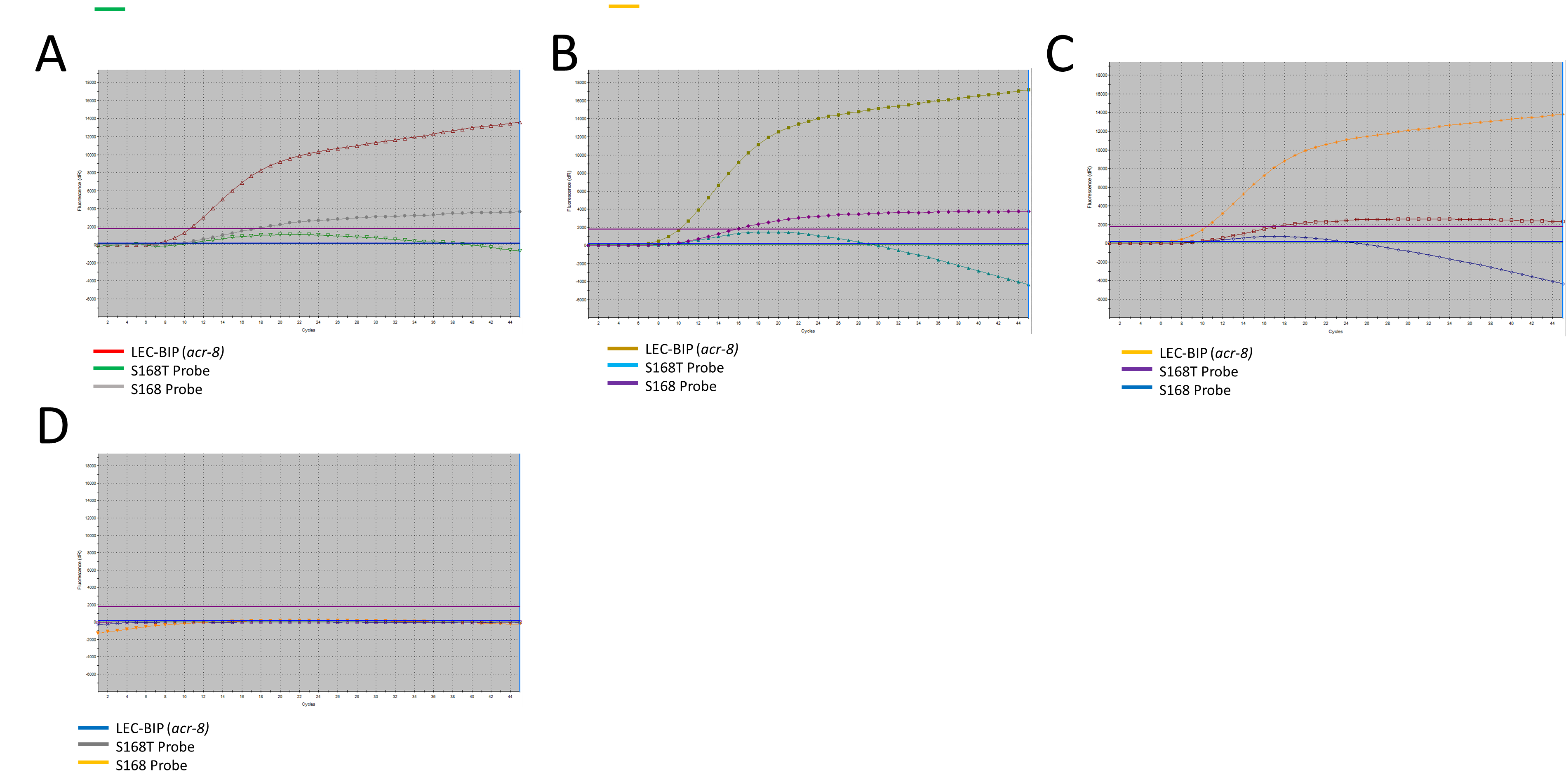


[Supplementary Figure 1: Multiplex S168T, S168, and *acr-8* LEC-LAMP challenged with mixed sample templates. Multiplex LEC-LAMP for the simultaneous detection of the *acr-8* gene, S168T and S168 alleles within a single tube assay at 63^o^C using generic *acr-8* probe, susceptible (S) and resistant probe (R). A: Detection of S168T, S168 alleles and *acr-8* in a single tube assay by modified resistant probe (R), susceptible probe (S), and LEC-BIP probe challenged with a 1:1 mix of the S168T:S168 templates**.** B: Detection of S168T, S168 alleles and *acr-8* in a single tube assay by modified resistant probe (R) susceptible probe (S) and LEC-BIP probe challenged with a 2:1 mix of the S168T:S168 template**.** C: Detection of S168T, S168 alleles and *acr-8* in a single tube assay by modified resistant probe (R) susceptible probe (S) and LEC-BIP probe challenged with a 1:2 mix of the S168T:S168 template**.** D: Comparison of low non-specific fluorescence signals of LEC-BIP, susceptible (S) and modified resistant probe (R) in the no template control sample.]
